# Cargoes of exosomes function as potential biomarkers for *Mycobacterium tuberculosis* infection

**DOI:** 10.3389/fimmu.2023.1254347

**Published:** 2023-10-18

**Authors:** Nan Wang, Yongliang Yao, Yingfen Qian, Dewen Qiu, Hui Cao, Huayuan Xiang, Jianjun Wang

**Affiliations:** ^1^ Department of Clinical Laboratory, Kunshan Hospital Affiliated to Jiangsu University, Suzhou, Jiangsu, China; ^2^ Department of Clinical Laboratory, Kunshan Fourth People’s Hospital, Suzhou, Jiangsu, China; ^3^ Department of Clinical Laboratory, Jiangxi Maternal and Child Health Hospital Maternal and Child Heath Hospital of Nanchang College, Nanchang, China; ^4^ Department of Food and Nutrition Safety, Jiangsu Provincial Center for Disease Control and Prevention, Nanjing, Jiangsu, China

**Keywords:** exosomes, mycobacterium tuberculosis, biomarkers, diagnosis, tuberculosis

## Abstract

Exosomes as double-membrane vesicles contain various contents of lipids, proteins, mRNAs and non-coding RNAs, and involve in multiple physiological processes, for instance intercellular communication and immunomodulation. Currently, numerous studies found that the components of exosomal proteins, nucleic acids or lipids released from host cells are altered following infection with *Mycobacterium tuberculosis*. Exosomal contents provide excellent biomarkers for the auxiliary diagnosis, efficacy evaluation, and prognosis of tuberculosis. This study aimed to review the current literatures detailing the functions of exosomes in the procedure of *M. tuberculosis* infection, and determine the potential values of exosomes as biomarkers to assist in the diagnosis and monitoring of tuberculosis.

## 1 Introduction

Tuberculosis (TB) is a bacterial infectious disease which causes a serious threat to the health and hygiene of human (1). According to the report of World Health Organization (WHO), ~25% of the worldwide population suffers from TB, and 1.6 million TB-related deaths occurred in 2021 (2). Notably, the incidence of TB among adolescents aged 10 to 24 years has increased in recent years (3). TB is transmitted via droplets of *Mycobacterium tuberculosis* (*M. tuberculosis*) complex when the body exhibits low levels of immunity (4). *M. tuberculosis* may infect various parts of the human, with the majority of bacteria colonizing the lungs (5). However, not all cases of *M. tuberculosis* infections will progress to TB, and the majority of infected individuals do not present with notable symptoms; a condition known as latent TB infection (LTBI) (6). Moreover, 5~10% of patients with LTBI develop active TB (ATB) during their whole lifetime; thus, presenting as novel sources of TB infection (7). This condition leads to complexities in the global prevention and control of TB.

*M. tuberculosis* enters the respiratory system, and is subsequently encapsulated by native immune cells, containing dendritic cells (DCs) and macrophages (8). Innate immune cells use membrane surface pattern recognition receptors (PRRs) to recognize the pathogen-associated molecular pattern (PAMP) or damage-associated molecular pattern (DAMP) of *M. tuberculosis*, and these trigger a signaling cascade within innate immune cells to induce the downstream immune response (9). Alveolar macrophages (AMs) are the primary targets of *M. tuberculosis* early infection (10). Phagocytosis of AMs is activated by the recognition of complement, Fcγ receptors, mannose receptor (11) or scavenger receptors (12), and rely on an intact surface sphingomyelin biosynthetic pathway to uptake *M. tuberculosis* into the cytoplasm to form phagosomes (13). During phagosome maturation, the pH value inside the phagosome decreases (14). Phagosomes bind to lysosomes to form phagolysosomes, which are further acidified, leading to *M. tuberculosis* inhibition or death (15). This process is known as LC3-associated phagocytosis (LAP). Macrophages also actively metabolize 1, 25-dihydroxy vitamin D (1, 25D) in response to the invasion of *M. tuberculosis*. 1, 25D participated in immune regulating responses through binding to the receptor of vitamin D, and regulating the expression of NOD2, antimicrobial proteins (CAMP and β-defensin 2) and inflammatory factors (IL-1β and IL-8). However, *M. tuberculosis* escapes the immune response via resisting the natural immunity of immune cells, and inhibiting apoptosis (16). Following the appearance of drug-resistant and multi-drug resistant *M. tuberculosis*, the diagnosis and therapy of TB have increased in complexity.

**Figure 1:**
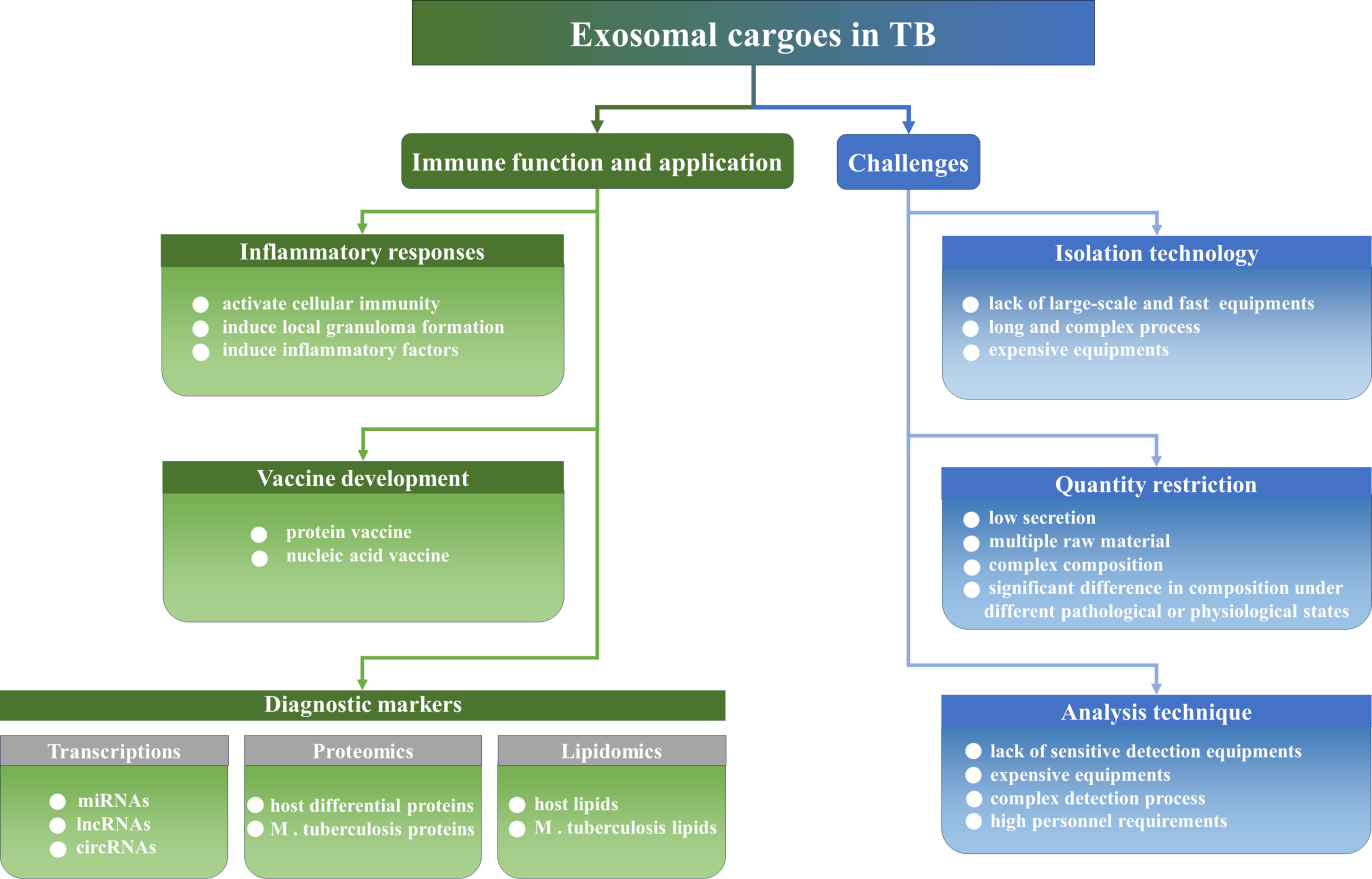
The immune function, the application and challenges of exosomes in TB. Exosomes regulate inflammatory responses and could be developed as vaccines and diagnostic biomarkers. Of course, exosomes still face a series of challenges to become a convenient diagnostic marker in clinical practice, such as isolation technologies, quantity limitations, and equipment and personnel limitations.

Therefore, the development of biomarkers with high specificity and sensitivity is particularly important for TB diagnosis. However, traditional methods for the etiologic diagnosis of TB, including sputum smears and culturing for *M. tuberculosis* exhibit limitations. *M. tuberculosis* cannot be distinguished from other acid-fast bacilli using sputum smears, and this method exhibits low levels of sensitivity. This limits the positive detection rate of patients with TB. Although culturing for *M. tuberculosis* is the common standard for ATB diagnosis, this method exhibits notable disadvantages. For example, *M. tuberculosis* culturing exhibits low positivity rates and prolonged culture times, which are not conducive to early diagnosis. X-ray imaging of the chest may aid in the detection of pulmonary TB; however, this process cannot be used to identify LTBI (17). Immunological strategies for TB diagnosis include tuberculin skin tests and INF-γ releasing assays. Notably, the aforementioned immunological tools are recommended for the diagnosis of *M. tuberculosis* infection; however, these are not currently recommended for ATB diagnosis (17, 18). Rapid molecular biology diagnostic techniques for TB, such as GeneXpert MTB/RIF and DNA sequencing, require high levels of instrumentation and specific expertise, and these techniques may lead to false negatives or false positives (19). At present, various studies is focused on the application of exosomes as biomarkers or vaccines for TB. Exosomes are stable structures with low invasiveness, which carry high levels of specific biomolecular information. The present article aimed to review the current literature detailing the immunomodulatory roles, diagnostic marker application of exosomes in the infection course of *M. tuberculosis*, and the challenges of exosomes as diagnostic markers for TB (**Figure 1**). The present review could provide a novel theoretical foundation for the role of exosomes as novel diagnostic markers of TB.

## 2 The biogenesis and functions of exosomes

Exosomes are nanovesicles that are with the diameter about 30~150 nm, and could be secreted into the extracellular matrix via numerous different cell types (20, 21). Exosomes form cup-shaped vesicles through endocytosis (20, 22, 23), including extracellular proteins and other components, and cell membrane receptors (23). These are known as early endosomes. The maturation of early endosomes into late endosomes [also known as multivesicular bodies (MVBs)] is accompanied by the sorting and enrichment of cargo molecules on early endosomal membranes, and the formation of intraluminal vesicles (ILVs) via membrane invagination (24). The mechanisms underlying MVB formation are categorized into endosomal sorting complexes required for transport (ESCRT)-dependent or independent pathways (25–27). Generated MVBs may fuse with lysosomes, and are degraded via lysosomal acid and proteolysis. MVBs may also fuse with the plasma membrane and secrete ILVs that are released to extracellular, or these directly bud through the cytoplasmic membrane to form exosomes (23–26). Notably, the inhibition of exosomes secretion leads to increased degradation of MVBs via lysosomes (26). The release of exosomes and their fusion with receptor cells is associated with the Ras superfamily. Rab proteins, including Rab 2B, 5A, 7, 9A, 11, 27 and 35 are molecular switches for the transport of MVBs, and these play critical functions in the process of vesicle transport (25, 27). Moreover, RalA/B GTPases promote the secretion of exosomes via the regulation of various effector proteins and lipids, such as phospholipase D1, which plays a role in the homeostasis of MVBs (28, 29), and PLD2, which is involved in the budding of exosomes cargoes (27). Rab GTPase facilitates the folding of membrane-bound soluble N-ethylmaleimide-sensitive factor attachment protein receptors into tetrameric coiled-coil complexes at exosomal and receptor cell membranes (30). This process is carried out via the recruitment of tethering proteins; thus, the two membranes remain in close proximity (31). Additionally, there are numerous other proteins in exosomes, such as the transmembrane 4 superfamily proteins (CD63, CD81 and CD9), flotillin, Alix and TSG101, which are also involved in exosomes biogenesis (27). The complex biogenesis, selection and transfer mechanisms contribute to the high heterogeneity of exosomes.

## 3 The functions of exosomes in *M. tuberculosis* infected hosts

Exosomes possess a wide range of various cargo molecules, including nucleic acids (miRNA, lncRNA, mRNA and DNA), proteins, lipids and metabolites (27, 32). Notably, exosomes are involved in intercellular messaging, maintenance of cellular homeostasis and immune regulatory processes. Results of previous studies demonstrated that the immune response induced by the interaction of exosomes with *M. tuberculosis* exerts an important impact on the development of TB (33). Intracellular *M. tuberculosis* uses SecA2 (34) and ESX-1 secretion systems to mediate cell membrane cleavage, and the *M. tuberculosis* genome, proteins and other components are transferred between cells via exosomes (35). Exosomes are recognized by PRRs as carriers of PAMP, which activate the inflammasome, LAP (34) and initiate an innate immune response for *M. tuberculosis* clearance (36). Exosomes released from *M. tuberculosis*-infected mesenchymal stem cells (MSCs) induce macrophages to produce TNF-α, C-C Motif Ligand-5 and iNOS. These factors promote inflammatory responses and immunoreaction through the signaling pathway synergistically mediated by Toll-like receptor 2/4 (TLR2/4) and MyD88 (37). Exosomes released from *M. tuberculosis-*infected macrophages induce the differentiation of naïve monocytes, and also activate MK-2 and NF-κb to produce functionally active macrophages (38). Following the stimulation of LPS and IFN-γ, exosomes released from macrophages bind to their secreted endoplasmic reticulum aminopeptidase 1 to enhance macrophage phagocytosis and NO synthesis activity (39). Necroptotic exosomes are phagocytosed by macrophages to induce the increased production of inflammatory cytokines, TNF-α, IL-6, and chemokine CCL2 (40). APCs secrete exosomes containing MHC-I/II that present antigenic information to T lymphocytes to activate specific immune responses (41, 42). Activated T cells stimulate DCs to increase the release of miR155-containing exosomes, further inducing specific T cell activation (43). Notably, T helper 1 (Th1) cells receive let-7b-containing exosomes released from Treg cells, and the inhibition of Th1 cell proliferation and IFN-γ secretion prevents excessive inflammatory injury (44). Exosomes released from activated T lymphocytes deliver genomic and mitochondrial DNA to DCs, which, in turn, trigger an innate immune response against *M. tuberculosis* infection (45), as the mitochondrial component is the main source of DAMPs (46). Exosomes may also stimulate autophagy and *M. tuberculosis* clearance (47). Exosomes derived from *M. tuberculosis-*infected neutrophils stimulate macrophage to produce O2- and induce autophagy, facilitating intracellular *M. tuberculosis* clearance (48).

**Figure 2:**
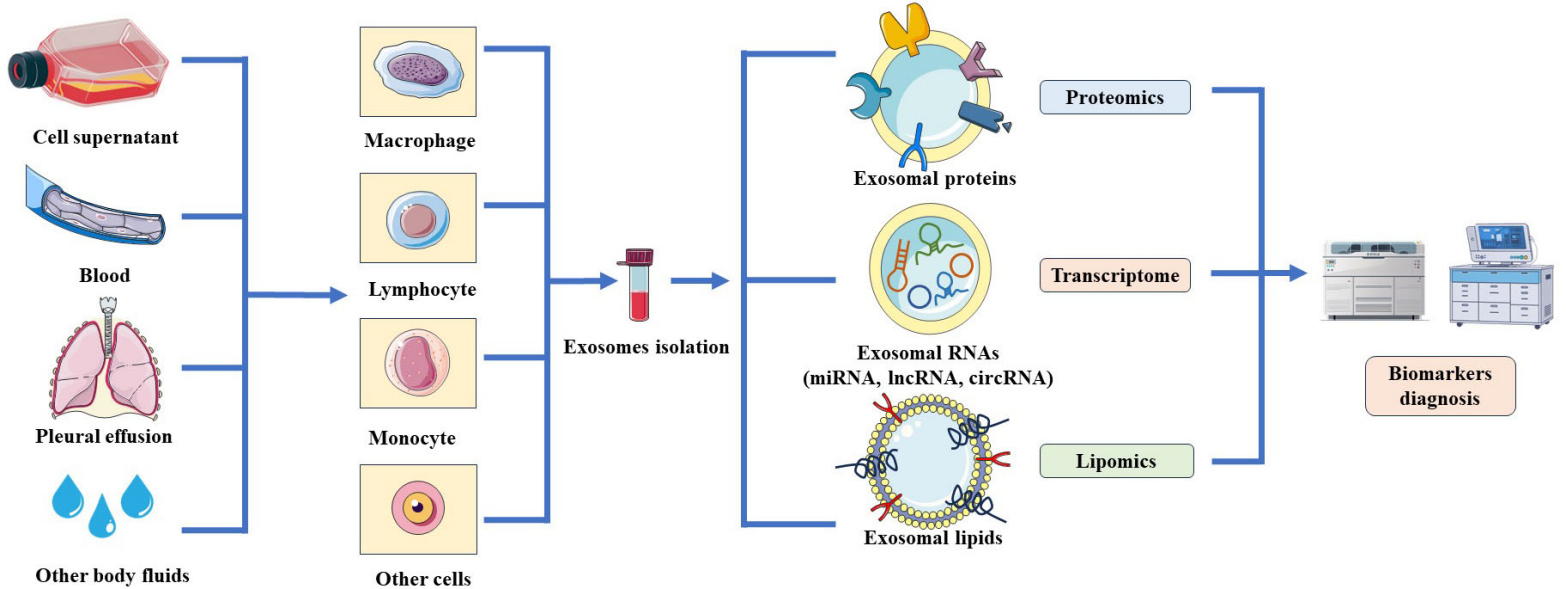
Schematic of the resources and analyzed technologies of exosomes. Exosomes are released by various cells such as macrophages, lymphocytes, monocytes and could purified from cell culture supernatant or body fluids. Exosomal contents could be screened through proteomics, transcriptomics and lipomics to identify potential biomarkers for the diagnosis of TB.

Although exosomes secreted by infected immune cells enhance the ability of uninfected immune cells to defend against *M. tuberculosis*, exosomes also aid *M. tuberculosis* immune evasion, providing a favorable environment for survival. Modified exosomes carry components of *M. tuberculosis* that affect the capacity of the host to eliminate them. Infected macrophages release exosomes containing miR-18a, which promotes *M. tuberculosis* survival in macrophages via inhibition of the autophagic process. This is carried out via regulation of the ATM-AMPK autophagic pathway (49). Exosomes derived from macrophages also inhibit CD4+ T cell antigen receptor signaling and IL-2 production (50), and downregulated IFN-γ induces the expression of CD64 or MHC-II in macrophages (51). Exosomes may exhibit a dual role in regulating the immune response. Exosomes come from a variety of tissues and cells, and with the rapid changes in new detection technologies, it has become possible for exosomes to become diagnostic biomarkers for TB (**Figure 2**).

## 4 Potential of exosomal miRNAs as biomarkers

### 4.1 The synthesis and function of miRNAs

**Figure 3:**
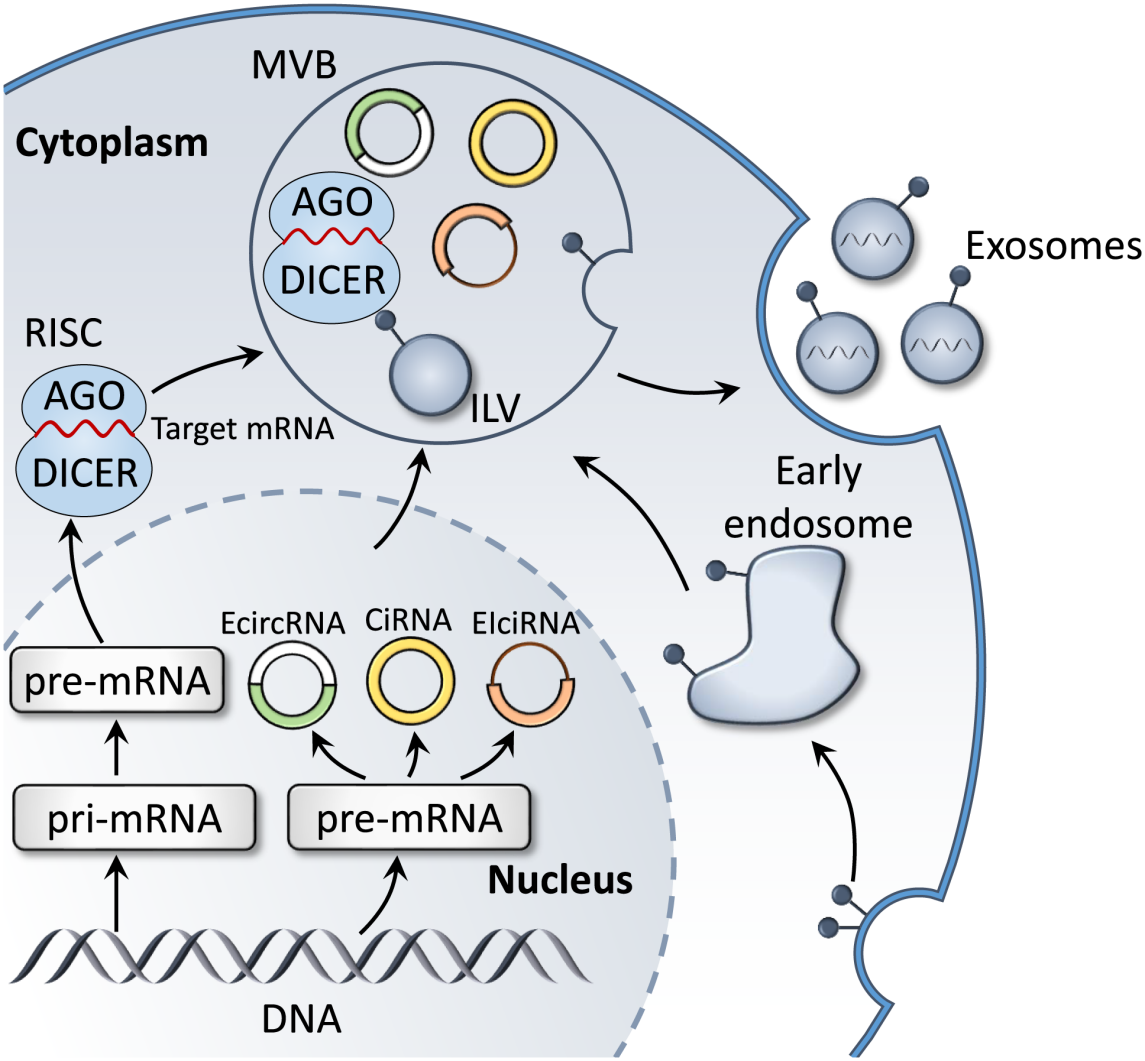
Biogenesis of exosomal miRNAs and circRNAs. In the cytoplasm, miRNA genes are transcribed into pri-miRNA, which is further processed to form pre-miRNA. Mature target miRNAs are integrated into RISC and fuse with MVBs, prior to releasing miRNA-containing exosomes. In addition, the main product of circRNA gene transcription, pre-mRNA, is processed to form three subclasses: ecircRNAs, EIciRNAs and circRNAs. These also fuse with MVB to form exosomes that are released into the extracellular environment.

MiRNAs are endogenous non-coding single-stranded RNA molecules that are 18-24 nucleotides in length, and are highly conserved during evolution (52). MiRNAs participate in regulating various fundamental biological functions, for instance cell proliferation, differentiation, migration (53), apoptosis (54) and autophagy (55), through binding to the 3’-untranslated region of target gene mRNAs (56, 57). The biosynthetic pathways of miRNAs could be classified into canonical and noncanonical pathways (56, 58). The canonical pathway is the dominant pathway for miRNA generation (57). The majority of miRNA genes are transcribed through RNA polymerase II in the nucleus to form pri-miRNAs containing hairpin structures (59). Subsequently, pri-miRNA is cleaved into pre-miRNA with stem-loop structures by the Drosha complex, which includes Drosha, RNase III, the double-stranded RNA-binding protein, DiGeorge syndrome critical region 8, and partner proteins (60). Thus, pre-miRNA is delivered into the cytoplasm via Exportin-5, and subsequently treated with RNase III endonuclease, Dicer, to produce double-stranded miRNAs (61). Double-stranded miRNAs and argonaute protein bind into the miRNA-induced silencing complex, where one strand is selected as the mature miRNA and the other strand is degraded (56, 61). Mature miRNAs may be packaged in exosomes and transferred between cells. As miRNAs are protected by the exosomal lipid bilayer, they may be protected from RNase degradation (**Figure 3**). Therefore, exosomal miRNAs remain highly stable, and remain in the blood and other bodily fluids for prolonged periods. Thus, these are considered as promising candidate biomarkers for TB.

### 4.2 The functions of exosomal miRNAs in *M. tuberculosis* infected subjects

Exosomal miRNAs released by macrophages infected with *M. tuberculosis* are stored in the supernatant, providing a theoretical basis for studying the potential of exosomal miRNAs as biomarkers for the diagnosis of *M. tuberculosis* infection. Zhang et al. showed that miR-20b-5p was expressed in exosomes from *M. tuberculosis-*infected macrophages, but not in exosomes from non-infected macrophages (62). Zhan et al. used high-throughput sequencing to detect miRNAs in exosomes secreted from *Mycobacterium bovis-*infected macrophages, and the results demonstrated that 20 exosomal miRNAs were increased, and 7 exosomal miRNAs were decreased in the infected group, compared with the non-infected group (63). Moreover, expression levels of let-7c-5p, miR-27-3p, miR-25-3p, let-7a-5p, miR-98-5p and miR-30a-3p were increased in the infected group, while the expression levels of miR-5110 and miR-194-5p were decreased (63). Results of a previous study suggested that the expression levels of exosomal miR-106a, miR-20a, miR-20b, miR-17 and miR-93 were downregulated in infected macrophages, as well as in the lungs, spleens and lymph nodes of mice infected with *M. tuberculosis* (64). The different exosomal miRNAs expression profiles of *M. tuberculosis*-infected patients were exhibited in body fluids. These miRNAs hold promise as potential biomarkers for the rapid and noninvasive diagnosis of TB. Kaushik et al. revealed that miR-185-5p in plasma exosomes were increased significantly in TB patients, compared with healthy controls (HCs), with a sensitivity and specificity of 50 and 93.75%, respectively. Moreover, Kaushik et al. suggested that the use of miR-185-5p in combination with other biomarkers may exhibit potential in TB diagnosis (65). Tu et al. confirmed that exosomal miR-423-5p is increased in the plasma of TB patients (66). The area under the curve (AUC) of the TB diagnostic model was 0.908 and the 10-fold cross validation demonstrated a prediction accuracy of 78.18%, which indicated that the model exhibited clinical value in differentiating ATB patients from HCs (65, 66). Lyu et al. demonstrated that miRNAs were differentially expressed in the serum of exosomes from HCs, LTBI patients and ATB patients, suggesting that miRNA cargo is selectively packaged into exosomes at different stages of *M. tuberculosis* infection (67). Notably, miR-450a-5p, let-7e-5p, miR-140-5p and let-7d-5p were only increased in the serum exosomes from LTBI patients, whereas miR-370-3p, miR-1246, miR-193b-5p, miR-2110 and miR-28-3p were only increased in the serum exosomes from patients with ATB (67). Moreover, miR-26a-5p was upexpressed in LTBI serum exosomes, but decreased in ATB (67). Results of further studies demonstrated that miR-140-3p, miR-423-3p and miR-3184-5p were sequentially increased in HCs, LTBI and ATB patients, and this differentiation may exhibit potential in determining the infectious stages of *M. tuberculosis* (67). In addition, Alipoor et al. demonstrated that the expression of miR-96, miR-484 and miR-425 were significantly increased in serum exosomes of TB patients, and the combined testing with sputum smears improved the detection rate of TB (68).

Exosomal miRNAs may also be used to differentiate TB from other lung-related diseases. Wang et al. verified the differential expression profiles of exosomal miRNAs in pleural effusions from adenocarcinoma of the lung (ADC), TB and other benign lesions using quantitative PCR (qPCR). Notably, the expression levels of miR-205-5p, miR-429, miR-483-5p, miR-375, miR-200b-3p and miR-200c-3p were higher in ADC-derived exosomes, compared with TB or other benign lesions (69). In addition, miR-148a-3p and miR-150-5p were upexpressed in TB-derived exosomes, and downexpressed in other benign lesion-derived exosomes. Interestingly, the opposite results were observed for the expression levels of miR-451a (69). Zhang et al. compared the expression profiles of exosomal miRNAs in TB pleural effusion and malignant pleural effusion. The results demonstrated that miR-3614-5p and miR-150-5p were decreased in malignant pleural effusion, and miR-629-5p, miR-200b-3p and miR-182-5p were increased in TB pleural effusion (70). Guio et al. carried out sRNA sequencing to analyze exosomes that were extracted from blood samples obtained from patients with LTBI, ATB or ADC. The results demonstrated that miR-210-3p and miR-143-3p were downregulated in the serum exosomes from patients with LTBI, and miR-20a-5p was upregulated in the serum exosomes from patients with LTBI (71). MiR-23b, miR-17 and miR-181b-5p were only downregulated in the serum exosomes from patients with ATB, and miR-584 was only upregulated in the serum exosomes from patients with ATB. A total of 15 miRNAs, including miR-320a, miR-185-5p, miR-144-3p, let-7f-5p and miR-199b-3p, were only downregulated in the serum exosomes of patients with ADC (71).

The diagnosis and treatment of drug-resistant TB (DR-TB) and multidrug-resistant TB (MDR-TB) are important for the prevention and control of TB. Notably, exosomal miRNAs exhibit potential as biomarkers in the early diagnosis and prognosis of DR-TB and MDR-TB. Carranza et al. analyzed the expression profiles of exosomal miRNAs in the serum of MDR-TB patients before and after 12 months of treatment, and revealed that the expression of exosomal miR-328-3p, miR-20a-3p and miR-195-5p and was decreased in the serum following treatment (72). Moreover, let-7e-5p and miR-197-3p were increased in post-treatment serum. Excluding patients with type 2 diabetes mellitus, results of the previous study demonstrated that the expression of let-7e-5p in the serum exosomes of patients with MDR-TB were upexpressed following treatment progression. Compared with HCs, miR-197-3p and miR-223-3p were decreased in the serum of DR-TB patients, while let7e-5p was increased in the serum of DR-TB patients (72). These results implied that the differential expression of exosomal miRNAs in the serum of MDR-TB patients with prolonged treatment may act as a biomarker for monitoring MDR-TB therapy, and that the differential expression in the serum of DR-TB and HCs may exhibit potential as a biomarker for determining drug-sensitive and drug-resistant TB.

In short, the differential expression profiles of miRNAs in TB patients may provide a novel perspective for the diagnosis and differential diagnosis of TB (**Table 1**). However, further investigations are still required to illustrate the mechanisms by which exosomal miRNAs contribute to the pathogenesis of TB, thus assisting in the development of biomarkers for the diagnosis and therapy of TB. The role of exosomal miRNAs in predicting the success of anti-TB therapy has also been highlighted in previous studies (73). Unfortunately, there is currently a limited amount of research focusing on the involvement of exosomes in TB prognosis, and additional investigations are needed to explore and understand the potential implications of exosomal miRNAs in TB prognosis.

**Table 1:** Summary of exosomal miRNAs from *M. tuberculosis* infected subjects.

Number	Exosomal miRNAs	Exosomes sources	Method screening	Expression pattern	Refs
1	miR-20b-5p	Supernatant of macrophage infected with *M. tuberculosis*	RT-PCR	decrease	(62)
2	miR-27-3p, let-7a-5p, let-7c-5p, miR-25-3p, miR-98-5p, miR-30a-3p, etc.	Supernatant of macrophage infected with *M. tuberculosis*	RNA sequencing	increase	(63)
3	miR-194-5p, miR-5110	Supernatant of macrophage infected with *M. bovis*	RNA sequencing	decrease	(63)
4	miR-185-5p	Plasma of TB patient	RNA sequencing	increase	(65)
5	miR-423-5p, miR-17-5p, miR-20b-5p	Serum of TB patient	RNA sequencing	increase	(66)
6	let-7e-5p, let-7d-5p, miR-450a-5p, miR-140-5p	Serum of LTBI patient	RNA sequencing	increase	(67)
7	miR-1246, miR-2110, miR-370-3p, miR -28-3p, miR-193b-5p, etc.	Serum of TB patient	RNA sequencing	increase	(67)
8	miR-26a-5p	Serum of ATB patient	RNA sequencing	decrease	(67)
9	miR-484, miR-425, miR-96, etc.	Serum of TB patient	qRT-PCR	increase	(68)
10	miR-205-5p, miR-200c-3p, miR-141-3p, etc.	Pleural effusion of TB patient	RNA sequencing	increase	(69)
11	miR-483-5p, miR-375	Pleural effusion of TB patient	RNA sequencing	decrease	(69)
12	miR-33a-3p, miR-153-3, miR-373-5p, etc.	Pleural effusion of TB patient	RNA sequencing	increase	(70)
13	miR-3120-5p, miR-489-3p, -miR-4669-5p, etc.	Pleural effusion of LTBI patient	sRNA sequencing	decrease	(70)
14	miR-143-3p, miR-210-3p, miR-20a-5p, etc.	Serum of LTBI patient	sRNA sequencing	increase	(71)
15	miR-23b, miR-17, miR-584, etc.	Serum of ATB patient	sRNA sequencing	increase	(71)

## 5 Exosomal circRNAs used as a biomarkers

### 5.1 The biogenesis and roles of circRNAs

Circular RNAs (circRNAs) are endogenous non-coding single stranded RNAs present in all eukaryotic cells (74), and are characterized by a covalently closed loop structure without a 5’ terminal cap and a 3’ terminal poly (A) tail (75). CircRNAs are grouped into intronic RNAs (ciRNAs), exonic circRNAs (ecircRNAs) and exon-intron circRNAs (elciRNAs) (76), displaying critical biological roles through playing as transcriptional regulators, ceRNA or miRNA sponges and protein templates (**Figure 3**) (77). Importantly, several studies have showed that the expression levels of circRNAs are dysregulated during *M. tuberculosis* infection (**Table 2**). CircRNAs are resistant to degradation by ribonucleases and RNA nucleic acid exonucleases due to their unique structure, and are highly conserved and detectable in various body fluids, such as plasma, saliva and urine. Additionally, circRNAs exhibit tissue specificity (84, 85); thus, are optimal candidates for the development of diagnostic biomarkers for clinical diseases.

**Table 2:** Summary of exosomal circRNAs in *M. tuberculosis* infected subjects.

Number	Exosomal circRNAs	Exosomes sources	Method screening	Expression pattern	Refs
1	circRNA_0001380	Plasma of ATB patient	qRT-PCR	decrease	(78)
2	circRNA_059914, circRNA_103017, circRNA_101128, etc.	Plasma of ATB patient	RNA sequencing	increase	(79)
3	circRNA_062400	Plasma of ATB patient	RNA sequencing	decrease	(79)
4	circRNA_103571, circRNA_091692, circRNA_102296, etc.	Plasma of ATB patient	circRNA microarrays	increase	(80)
5	circRNA_103571, circRNA_406755	Plasma of ATB patient	circRNA microarrays	decrease	(80)
6	circRNA_0009024, circRNA_0001953, circRNA_0008297, etc.	Plasma of ATB patient	RNA sequencing	increase	(81)
7	circRNA_0001204, circRNA_0001747	Plasma of ATB patient	RNA sequencing	decrease	(82)
8	circRNA_051239, circRNA_029965, circRNA_404022, etc.	Serum of ATB patient	RNA sequencing	increase	(83)

### 5.2 The functional analysis of exosomal circRNAs in samples of TB patients

Yuan et al. used bioinformatics to screen three central genes related to the development of TB, including circRNA_0002419 and circRNA_0007919 (86). The aforementioned genes were upregulated in TB tissues, and circRNA_0005521 was decreased in TB tissues (86). Moreover, Yi et al. confirmed that both miR-223-3p and miR-448 were decreased in the plasma of patients with TB, and also concluded that the mRNA-miRNA-circRNA interaction chain may function significant roles in *M. tuberculosis* infection (87). In addition, SAMD8_circRNA_994 and TWF1_circRNA_9897 may act as novel diagnostic biomarkers for TB (87). Zhang et al. carried out qPCR and demonstrated that circRNA_0028883 expression levels were upexpressed in PBMCs from ATB patients (88). Moreover, Zhang et al. performed ROC curve analysis and determined an AUC value of 0.773 (88). These foundings suggested that circRNA_0028883 could serve as a novel biomarker for ATB diagnosis. Further studies demonstrated that compared with HCs, circRNA_0001380 was decreased significantly in PBMCs from ATB patients (78), and circRNA_0009128 or circ_0005836 were also downexpressed in PBMCs of ATB patients (89). CircRNA_101128, circRNA_059914 and circRNA_103017 were expressed at higher levels in PMBCs from ATB patients, while circRNA_062400 expression was significantly lower in ATB samples than in HCs (79). The expression of circRNA_103571 decreased in the plasma of ATB patients, and this study demonstrated an interaction between circRNA_103571 and ATB-associated miRNAs (miR-29a and miR-16) (80). Thus, the selective expression of exosomal circRNA in TB demonstrates that exosomes exhibit potential as non-invasive diagnostic tools.

Huang et al. reported that circRNA_001937, circRNA_005086 and circRNA_009024 increased significantly, but circRNA_102101, circRNA_104296and circRNA_104964 decreased obviously in PBMCs of ATB patients, compared with HCs (90). In addition, circRNA_001937 expression levels were markedly increased in PBMCs of ATB patients, compared with patients with pneumonia, lung cancer and chronic obstructive pulmonary disease. Interestingly, circRNA_001937 could be increased following ATB treatment (90). Results of this study further demonstrated that circRNA_0003528, circRNA_0009024, circRNA_0001953, circRNA_0003524, circRNA_0008297 and circRNA_0015879 in plasma were increased markedly in ATB patients. However, the expression levels of circRNA_0001747and circRNA_0001204 were notably decreased in the plasma of ATB patients, compared with those of HCs (81). Reports also show that circRNA_0009024 and circRNA_0001953 in plasma were associated with the severity of ATB disease. Moreover, the AUC value of the ROC curve of ATB patients was increased to 0.928 with the combined detection of circRNA_0001747 and circRNA_0001204, and in ATB patients, the expression levels of circRNA_0001747and circRNA_0001204 returned to baseline in the plasma following treatment (82). Huang et al. also reported that monocyte derived macrophages from ATB patients exhibited significantly higher levels of circRNA_0043497 compared with HCs, with an AUC value of 0.860 (91). In addition, circRNA_0043497 levels decreased and returned to baseline following anti-TB therapy (91). Therefore, circRNAs may be used for the differential diagnosis of TB and associated diseases, and for the assessment of TB severity and prognosis. The combined detection of multiple circRNAs exhibited greater diagnostic value for patients with TB. CircRNA may also aid in distinguishing patients with DR-TB from patients with pan-sensitive TB. Liu et al. revealed that circRNA_051239, circRNA_404022 and circRNA_029965 were increased in the sera of ATB patients, and circRNA_051239 was decreased significantly in the sera of patients with DR-TB (83).

CircRNAs are highly enriched in exosomes compared with production cells. The regulation of relevant miRNAs in donor cells causes to changes in the composition of exosomal circRNAs and may transmit molecular information to recipient cells (92). In this process, various RNA binding proteins act as key factors that facilitate the propagation of circRNAs in donor cells (93). Results of a previous study demonstrated that exosomal circRNAs of host cells exhibit distinct expression patterns following *M. tuberculosis* infection (65). This provides evidence for the potential of exosomal circRNAs as biomarkers for the diagnosis of TB. But there is still a need for large-scale screening of blood samples, and further investigation based on existing research is required to explore the potential role of exosomal circRNAs as biomarkers for early diagnosis and prognosis of TB.

## 6 Exosomal proteins act as biomarkers of TB

At present, studies is focused on the protein content of exosomes. Previous studies have demonstrated which exosomes from *M. tuberculosis-*infected macrophages are present with highly antigenic mycobacterial proteins, such as KatG (Rv1908c), GroES (Rv3418c), GlnA (Rv2220), MPT63 (Rv1926c), ESAT-6 (Rv3875), 19 KDa lipoprotein/LpqH (Rv3763), CFP-10, Ag85 complex (Rv3804c, Rv1886c, Rv0129c) and SodA (Rv3846) (94). Lee et al. performed proteomic analysis of *M. tuberculosis* extracellular vehicles (EVs) and identified a total of 287 vesicular proteins (95). Among them, SodB, PstS1, EsxN, KatG, LppX, Apa, LpqH, FadA3, GlnA1, AcpM, FbpA, Mtc28 and Fba were abundant proteins in EVs of *M. tuberculosis*. Proteins such as SodB, FbpA, LpqH, FbpC, FbpB, and PstS1 were associated with *M. tuberculosis* virulence (95). The aforementioned *M. tuberculosis* proteins carried by exosomes may impact the innate or adaptive immune response (96), and may play important functions in the development of TB.

**Figure 4:**
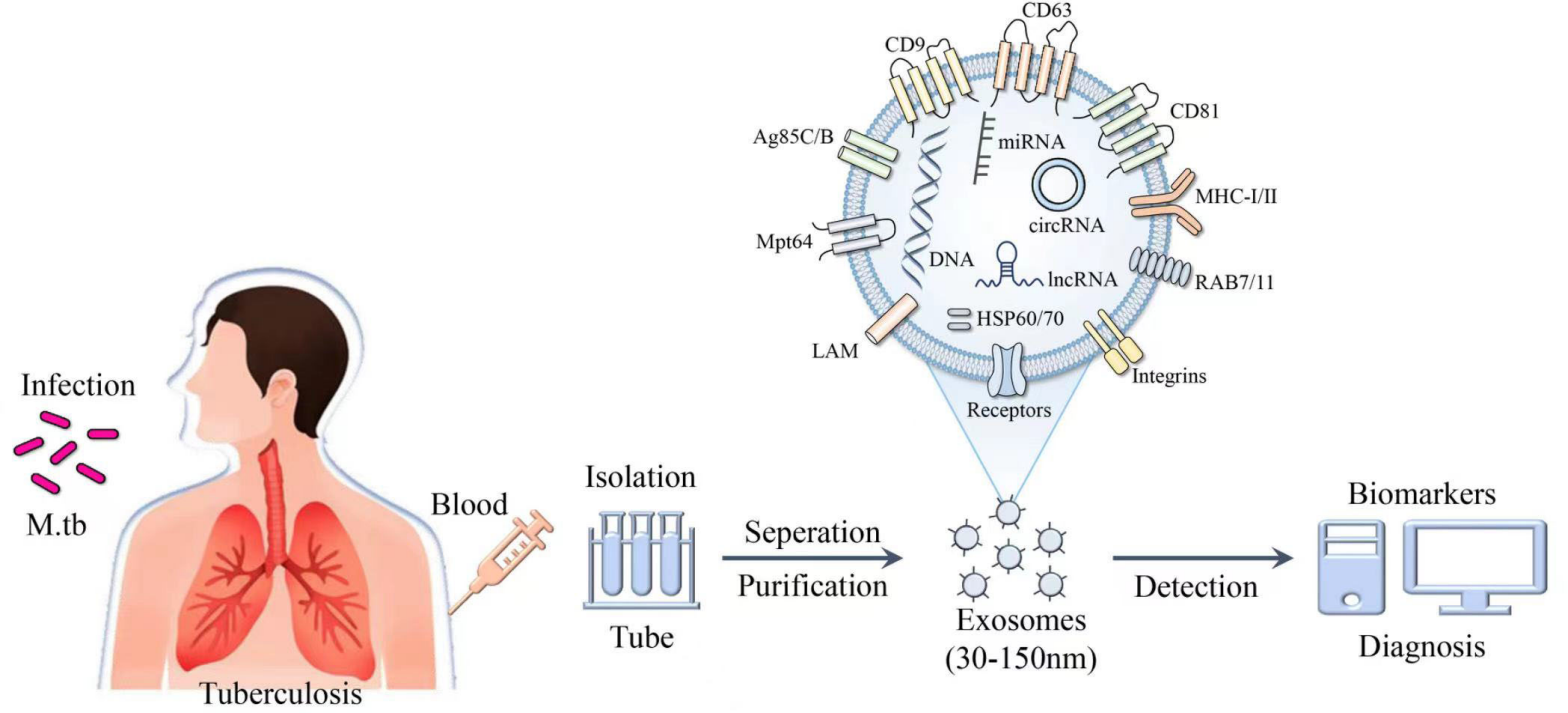
Schematic of the composition and identification of exosomes. Exosomes purified from the blood of patients with *M. tuberculosis* contain a variety of derivatives, such as nucleic acids, proteins and lipids, and exhibit potential as biomarkers in the diagnosis of TB.

The composition of exosomal proteins released by cells infected with *M. tuberculosis* is altered (**Table 3**), thus the differential expression profiles of proteins in TB patients may provide a novel perspective for the diagnosis of *M. tuberculosis* infection (**Figure 4**). Diaz et al. evaluated differences in exosomal proteins between *M. tuberculosis*-infected and -uninfected macrophages using tandem mass spectrometry. Results of study demonstrated that a total of 41 proteins were significantly upregulated in the exosomes of *M. tuberculosis*-infected cells (97). Notably, some of the aforementioned proteins were confirmed via western blot analysis, including moesin, HSP90, vimentin and Coronin 1C (97). Kruh-Garcia et al. highlighted bacterial-derived biomarkers in the serum exosomes of TB patients, including multiple peptides from 8 proteins (Antigen85B, Antigen85C, Apa, HspX, BfrB, Mpt64, GlcB and KatG). Of these, 29 peptides from 17 proteins were unique to ATB patients, such as AcpM, Ald, Ag85a, DnaK, Mpt51, GroES, Mpt63, Mpt53 and MrsA (98). Among 41 patients with TB, biomarker candidates consisting of seven peptides were used to correctly diagnose 83% of TB cases, and at least one peptide was present in 81% of TB patients, and 90% of patients with extrapulmonary TB (98). The combined testing of two peptides increased the diagnosis of patients with intrapulmonary or extrapulmonary TB to 90%. Obviously, human immunodeficiency virus infection does not affect the number of peptides observed in the plasma of TB patients (98). These results demonstrated that exosomal proteins may be used as biomarkers for TB diagnosis, and that the simultaneous detection of multiple peptides may substantially improve the accuracy of TB diagnosis. Through proteomic analysis, Zhang et al. indicated 123 differential proteins in serum exosomes from HCs and ATB patients, including 40 upregulated proteins and 83 downregulated proteins (99). Notably, lipopolysaccharide binding protein expression was increased in the serum exosomes of ATB patients, while CD36 and MHC-I expression levels were decreased (99). The aforementioned three proteins were identified as potential biomarkers for ATB diagnosis with ROC analysis. In addition, Mehaffy et al. characterized peptides from *M. tuberculosis* proteins involved in nitrogen metabolism, and these included GarA (Rv1827), peptide FLL and SVF belonging to glutamine synthetase GlnA1 (Rv2220) (100). Heat shock chaperone proteins, including GroES and DnaK (Rv0350) were also characterized in the serum EVs of patients with LTBI (100). Among them, a single peptide in glutamine synthetase (GlnA1) enzyme was present in the serum of 82% of LTBI patients, indicating that peptides from *M. tuberculosis* proteins involved in nitrogen metabolism may act as candidate biomarkers for the detection of LTBI pathogen specificity (100). Exosomal proteins may be used to distinguish ATB from other associated diseases. Results of previous studies demonstrated that Hsp16.3 protein levels were detected in exosomes extracted from the plasma of ATB patients; however, Hsp16.3 was not detected in the plasma exosomes of LTBI patients (101). Biadglegne et al. demonstrated that haptoglobin (HP), proteoglycan 4, CD151, stomatin, ICAM-2, alpha-1-acid glycoprotein 1, solute carrier family 2A3 and serum amyloid A-1 protein were abundant in plasma exosomes from TB patients, compared with HCs (102). In addition, immunoglobulins, glutamate receptor-interacting protein 1 and complement component 1r were enriched in TB patients’ lymphadenitis (102). Thus, the specific expression levels of exosomal proteins in TB and TB lymphadenitis may exhibit potential for diagnosis and differential diagnosis.

**Table 3:** Summary of exosomal proteins and lipids from *M. tuberculosis* infected subjects.

Number	Exosomal proteins	Exosomal lipids	Exosomes sources	Method screening	Expression pattern	Refs
1	HSP90, vimentin, Coronin 1 C, moesin, etc	—	Supernatant of macrophage infected with *M. tuberculosis*	Tandem mass spectrometry	increase	(97)
2	AcpM, Ag85a, Ald, DnaK, GroES, Mpt51, Mpt53, Mpt63, MrsA, etc	—	Serum of ATB patient	MRM-MS	increase	(98)
3	LBP	—	Serum of ATB patient	ELISA	increase	(99)
4	CD36, MHC-I	—	Serum of ATB patient	ELISA	decrease	(99)
5	Rv1827, Rv2220, Rv0350, etc.	—	Serum of LTBI patient	MRM-MS	increase	(100)
6	Hsp16.3		Plasma of ATB patient	Western blot	increase	(101)
7	HP, PRG4, STOM, CD151, ICAM2, ORM1, SAA1, SLC2A3, etc.	—	Plasma of TB patient	Tandem mass Tag-labeled (TMT)	increase	(102)
8	C1R, GRIP1	—	Plasma of TB patient	TMT	increase	(102)
9	—	PS	Supernatant of macrophage infected with *M. tuberculosis*	Western blotting	increase	(103)
10	—	LAM, CFP-10	Urine of TB patient	I-PCR	increase	(104)
11	—	TAG, CEs	Plasma of TB patient	ESI-MS	increase	(102)

Exosomal proteins exhibit potential in determining the prognosis of TB patients. Du et al. confirmed that S100A9 and C4BPA in plasma exosomes of LTBI patients were differentially decreased following therapy, and the area under the ROC curve was 0.73 and 0.69, respectively (105). Biadglegne et al. reported that plasma exosomes myosin-9, IG chain IGHV4-28 and GRIP1 were increased markedly in TB patients following anti-TB treatment, while HP, ficolin 3, transmembrane protein 215, serum amyloid A-4 protein and apolipoprotein B-100 were decreased following anti-TB treatment (102).

Due to their small size, exosomes pass freely across the tissue barriers of the body. Exosomes protect proteins from free protease hydrolysis using their lipid bilayer membrane structure (106). The composition of exosomal proteins from infected *M. tuberculosis* reflects the exosomal proteomic profile more directly than that of nucleic acids (106, 107). In conclusion, exosomal proteins may exhibit potential as novel biomarkers of TB, and could be used for the development of new diagnostic methods. In addition, lipids and lipid metabolism are currently a research hotspot, and exosomes lipids are also potential diagnostic biomarkers for tuberculosis.

## 7 Exosomal lipids function as biomarkers of TB

The lipid components of the host is closely associated with the pathogenic mechanisms of *M. tuberculosis*. When macrophages consume glucose, *M. tuberculosis* could utilize host lipids as the main source of energy (108). *M. tuberculosis* may also produce a variety of unique lipids that act as inflammatory regulators, and these are implicated in preventing phagosome maturation (109). A previous study revealed that lipids produced by *M. tuberculosis* are glycolipids, including atrehalose-6, 6’-dimycolate, lipomannan, lipoarabinomannan (LAM) and phosphatidylinositol mannosides (PIMs), the sugar fraction of which is recognized by PRRs that stimulate the innate immune response of the organism during infection (110). In summary, *M. tuberculosis* lipids take a multifaceted approach to disrupt the antimicrobial response of host cells to ensure their survival and proliferation in host cells, and also act important roles in the immune process as immunomodulators.

Existing studies have shown that exosomes in the peripheral blood of TB patients contain rich lipids, with various sources and components. These liposomes can be used to assess TB infection and may serve as biomarkers for TB diagnosis (**Table 3**). Garcia-Martinez et al., discovered that phosphatidylserine (PS) was more abundant in extracellular vesicles released from macrophages of *M. tuberculosis*-infected mouse compared to those of normal mouse (103). Dahiya et al. detected LAM and CFP-10, using immunopolymerase chain reaction in urine EVs from patients with pulmonary and extrapulmonary TB (104). Apparently, the sensitivity of LAM detection in the urine EVs of patients with pulmonary and extrapulmonary TB was 74.3 and 67.9%, respectively, and the specificity was 91.5-92.8% (104). The presence of large amounts of triacylglycerols and cholesterylesters (CEs) in plasma exosomes of patients infected with *M. tuberculosis* has also been reported, while CEs are difficult to detect in HCs (102). The accumulation of CEs facilitates the survival and multiplication of *M. tuberculosis*, and promotes the dissemination of *M. tuberculosis* following cytolytic disintegration (102). Han et al. revealed that plasma CEs may act as novel biomarkers in TB diagnosis with optimal accuracy (AUC, 0.863; specificity, 83.5%; sensitivity, 79.4%) (111). Thus, certain differentially expressed lipid components in exosomes may also play a role in TB diagnosis. However, there is currently relatively little research on this topic, and there are also relatively few lipids found to have diagnostic value.

## 8 Future perspective

Exosomes have vast clinical potential in the diagnosis of TB and the differential diagnosis of related diseases (112, 113). However, the current research on exosomes in the prognosis and therapeutic evaluation of TB is relatively limited. In order to fully explore the potential of exosomes as biomarkers for TB (114), it is necessary to collect more clinical samples, conduct large-scale clinical trials, and utilize highly sensitive and specific techniques to analyze and identify the changes in exosomal components after *M. tuberculosis* infection (115). Unfortunately, obtaining highly pure exosomes remains technical challenges for large-scale clinical diagnostic applications due to the lack of standardized isolation and purification protocols and the high heterogeneity of exosomes (116). Therefore, it is necessary to conduct in-depth research to innovate and improve exosomes extraction techniques, in order to provide more accurate and reliable methods for the diagnosis, treatment, and monitoring of TB in the future.

## Author contributions

NW: Conceptualization, Writing – original draft. YY: Conceptualization, Visualization, Writing – review & editing. YQ: Investigation,Visualization, Validation, Writing– review & editing. DQ: Formal Analysis, Investigation, Methodology, Resources, Validation, Writing – review & editing. HC: Formal Analysis, Investigation, Methodology, Resources, Writing – review & editing. HX: Data curation, Investigation, Resources, Writing – review & editing. JW: Conceptualization, Funding acquisition, Project administration, Validation, Writing – review & editing.

## References

[1] Drain PK Bajema KL Dowdy D Dheda K Naidoo K Schumacher SG. Incipient and subclinical tuberculosis: a clinical review of early stages and progression of infection. Clin Microbiol Rev (2018) 31(4):e00021–18. doi: 10.1128/CMR.00021-18 PMC6148193 30021818

[2] Bagcchi S. WHO’s global tuberculosis report 2022. Lancet Microbe (2023) 4(1):e20. doi: 10.1016/S2666-5247(22)00359-7 36521512

[3] Snow KJ Cruz AT Seddon JA Ferrand RA Chiang SS Hughes JA. Adolescent tuberculosis. Lancet Child Adolesc Health (2020) 4(1):68–79. doi: 10.1016/S2352-4642(19)30337-2 31753806 PMC7291359

[4] Moule MG Cirillo JD. *Mycobacterium tuberculosis* dissemination plays a critical role in pathogenesis. Front Cell Infect Microbio (2020) 10:65. doi: 10.3389/fcimb.2020.00065 PMC7053427 32161724

[5] Suárez I Fünger SM Kröger S Rademacher J Fätkenheuer G Rybniker J. The diagnosis and treatment of tuberculosis. Dtsch Arztebl Int (2019) 116(43):729–35. doi: 10.3238/arztebl.2019.0729 31755407

[6] Carranza C Pedraza-Sanchez S de Oyarzabal-Mendez E Torres M. Diagnosis for latent tuberculosis infection: new alternatives. Front Immunol (2020) 11:2006. doi: 10.3389/fimmu.2020.02006 33013856 PMC7511583

[7] Ferluga J Yasmin H Al-Ahdal MN Bhakta S Kishore U. Natural and trained innate immunity against Mycobacterium tuberculosis. Immunobiology (2020) 225(3):151951. doi: 10.1016/j.imbio.2020.151951 32423788

[8] Sia JK Rengarajan J. Immunology of mycobacterium tuberculosis infections. Microbiol Spectr (2019) 7(4):10. doi: 10.1128/microbiolspec.GPP3-0022-2018 PMC6636855 31298204

[9] Boom WH Schaible UE Achkar JM. The knowns and unknowns of latent *Mycobacterium tuberculosis* infection. J Clin Invest (2021) 131(3):e136222. doi: 10.1172/JCI136222 33529162 PMC7843221

[10] Cohen SB Gern BH Delahaye JL Adams KN Plumlee CR Winkler JK. Alveolar macrophages provide an early *mycobacterium tuberculosis* niche and initiate dissemination. Cell Host Microbe (2018) 24(3):439–446.e4. doi: 10.1016/j.chom.2018.08.001 30146391 PMC6152889

[11] Choudhuri S Chowdhury IH Garg NJ. Mitochondrial regulation of macrophage response against pathogens. Front Immunol (2021) 11:622602. doi: 10.3389/fimmu.2020.622602 33679710 PMC7925834

[12] Linares-Alcántara E Mendlovic F. Scavenger receptor A1 signaling pathways affecting macrophage functions in innate and adaptive immunity. Immunol Invest (2022) 51(6):1725–55. doi: 10.1080/08820139.2021.2020812 34986758

[13] Niekamp P Guzman G Leier HC Rashidfarrokhi A RiChina V Pott F. Sphingomyelin Biosynthesis Is Essential for Phagocytic Signaling during *Mycobacterium tuberculosis* Host Cell Entry. mBio (2021) 12(1):e03141–20. doi: 10.1128/mBio.03141-20 PMC7858061 33500344

[14] Rai R Singh V Mathew BJ Singh AK Chaurasiya SK. Mycobacterial response to an acidic environment: protective mechanisms. Pathog Dis (2022) 80(1):ftac032. doi: 10.1093/femspd/ftac032 35953394

[15] Weiss G Schaible UE. Macrophage defense mechanisms against intracellular bacteria. Immunol Rev (2015) 264(1):182–203. doi: 10.1111/imr.12266 25703560 PMC4368383

[16] Wang J Wang Y Tang L Garcia RC. Extracellular vesicles in mycobacterial infections: Their potential as molecule transfer vectors. Front Immunol (2019) 10:1929. doi: 10.3389/fimmu.2019.01929 31474995 PMC6703136

[17] Acharya B Acharya A Gautam S Ghimire SP Mishra G Parajuli N. Advances in diagnosis of Tuberculosis: an update into molecular diagnosis of Mycobacterium tuberculosis. Mol Biol Rep (2020) 47(5):4065–75. doi: 10.1007/s11033-020-05413-7 32248381

[18] Halliday A Masonou T Tolosa-Wright M Mandagere V Lalvani A. Immunodiagnosis of active tuberculosis. Expert Rev Respir Med (2019) 13(6):521–32. doi: 10.1080/17476348.2019.1615888 31134820

[19] MacLean E Kohli M Weber SF Suresh A Schumacher SG Denkinger CM. Advances in molecular diagnosis of tuberculosis. J Clin Microbiol (2020) 58(10):e01582–19. doi: 10.1128/JCM.01582-19 PMC7512154 32759357

[20] Kang T Atukorala I Mathivanan S. Biogenesis of extracellular vesicles. Subcell Biochem (2021) 97:19–43. doi: 10.1007/978-3-030-67171-6_2 33779912

[21] Li YJ Wu JY Wang JM Hu XB Xiang DX. Emerging strategies for labeling and tracking of extracellular vesicles. J Control Release (2020) 328:141–59. doi: 10.1016/j.jconrel.2020.08.056 32882270

[22] Dreyer F Baur A. Biogenesis and functions of exosomes and extracellular vesicles. Methods Mol Biol (2016) 1448:201–16. doi: 10.1007/978-1-4939-3753-0_15 27317183

[23] Kalluri R LeBleu VS. The biology, function, and biomedical applications of exosomes. Science (2020) 367(6478):eaau6977. doi: 10.1126/science.aau6977 32029601 PMC7717626

[24] Kita S Shimomura I. Extracellular vesicles as an endocrine mechanism connecting distant cells. Mol Cells (2022) 45(11):771–80. doi: 10.14348/molcells.2022.0110 PMC9676990 36380729

[25] Rayamajhi S Aryal S. Surface functionalization strategies of extracellular vesicles. J Mater Chem B (2020) 8(21):4552–69. doi: 10.1039/D0TB00744G 32377649

[26] Eitan E Suire C Zhang S Mattson MP. Impact of lysosome status on extracellular vesicle content and release. Ageing Res Rev (2016) 32:65–74. doi: 10.1016/j.arr.2016.05.001 27238186 PMC5154730

[27] Gurunathan S Kang MH Kim JH. A comprehensive review on factors influences biogenesis, functions, therapeutic and clinical implications of exosomes. Int J Nanomed (2021) 16:1281–312. doi: 10.2147/IJN.S291956 PMC7898217 33628021

[28] Zago G Biondini M Camonis J Parrini MC. A family affair: A Ral-exocyst-centered network links Ras, Rac, Rho signaling to control cell migration. Small GTPases (2019) 10(5):323–30. doi: 10.1080/21541248.2017.1310649 PMC6748358 28498728

[29] Ghoroghi S Mary B Larnicol A Asokan N Klein A Osmani N. Ral GTPases promote breast cancer metastasis by controlling biogenesis and organ targeting of exosomes. Elife (2021) 10:e61539. doi: 10.7554/eLife.61539 33404012 PMC7822591

[30] Wickner W Rizo J. A cascade of multiple proteins and lipids catalyzes membrane fusion. Mol Biol Cell (2017) 28(6):707–11. doi: 10.1091/mbc.e16-07-0517 PMC5349777 28292915

[31] Borchers AC Langemeyer L Ungermann C. Who’s in control? Principles of Rab GTPase activation in endolysosomal membrane trafficking and beyond. J Cell Biol (2021) 220(9):e202105120. doi: 10.1083/jcb.202105120 34383013 PMC8366711

[32] Kugeratski FG Hodge K Lilla S McAndrews KM Zhou X Hwang RF. Quantitative proteomics identifies the core proteome of exosomes with syntenin-1 as the highest abundant protein and a putative universal biomarker. Nat Cell Biol (2021) 23(6):631–41. doi: 10.1038/s41556-021-00693-y PMC9290189 34108659

[33] Schorey JS Bhatnagar S. Exosome function: from tumor immunology to pathogen biology. Traffic (2008) 9(6):871–81. doi: 10.1111/j.1600-0854.2008.00734.x PMC3636814 18331451

[34] Cheng Y Schorey JS. Extracellular vesicles deliver Mycobacterium RNA to promote host immunity and bacterial killing. EMBO Rep (2019) 20(3):e46613. doi: 10.15252/embr.201846613 30683680 PMC6399609

[35] Tiwari S Casey R Goulding CW Hingley-Wilson S Jacobs WR Jr. Infect and inject: how *mycobacterium tuberculosis* exploits its major virulence-associated type VII secretion system, ESX-1. Microbiol Spectr (2019) 7(3):10. doi: 10.1128/microbiolspec.BAI-0024-2019 PMC6698389 31172908

[36] Chen Z Larregina AT Morelli AE. Impact of extracellular vesicles on innate immunity. Curr Opin Organ Transplan (2019) 24(6):670–8. doi: 10.1097/MOT.0000000000000701 PMC7328797 31592838

[37] Liu M Wang Z Ren S Zhao H. Exosomes derived from mycobacterium tuberculosis-infected MSCs induce a pro-inflammatory response of macrophages. Aging (Albany NY) (2021) 13(8):11595–609. doi: 10.18632/aging.202854 PMC8109131 33872217

[38] Singh A Das K Banerjee S Sen P. Elucidation of the signalling pathways for enhanced exosome release from *Mycobacterium*-infected macrophages and subsequent induction of differentiation. Immunology (2023) 168(1):63–82. doi: 10.1111/imm.13561 36240165

[39] Goto Y Ogawa Y Tsumoto H Miura Y Nakamura TJ Ogawa K. Contribution of the exosome-associated form of secreted endoplasmic reticulum aminopeptidase 1 to exosome-mediated macrophage activation. Biochim Biophys Acta Mol Cell Res (2018) 1865(6):874–88. doi: 10.1016/j.bbamcr.2018.03.009 29567213

[40] Shlomovitz I Erlich Z Arad G Edry-Botzer L Zargarian S Cohen H. Proteomic analysis of necroptotic extracellular vesicles. Cell Death Dis (2021) 12(11):1059. doi: 10.1038/s41419-021-04317-z 34750357 PMC8575773

[41] André F Chaput N Schartz NE Flament C Aubert N Bernard J. Exosomes as potent cell-free peptide-based vaccine. I. Dendritic cell-derived exosomes transfer functional MHC class I/peptide complexes to dendritic cells. Immunol (2004) 172(4):2126–36. doi: 10.4049/jimmunol.172.4.2126 14764678

[42] Ramachandra L Qu Y Wang Y Lewis CJ Cobb BA Takatsu K. *Mycobacterium tuberculosis* synergizes with ATP to induce release of microvesicles and exosomes containing major histocompatibility complex class II molecules capable of antigen presentation. Infect Immun (2010) 78(12):5116–25. doi: 10.1128/IAI.01089-09 PMC2981298 20837713

[43] Okoye IS Coomes SM Pelly VS Czieso S Papayannopoulos V Tolmachova T. MicroRNA-containing T-regulatory-cell-derived exosomes suppress pathogenic T helper 1 cells. Immunity (2014) 41(1):89–103. doi: 10.1016/j.immuni.2014.05.019 25035954 PMC4104030

[44] Lindenbergh MFS Koerhuis DGJ Borg EGF van ‘t Veld EM Driedonks TAP Wubbolts R. Bystander T-cells support clonal T-cell activation by controlling the release of dendritic cell-derived immune-stimulatory extracellular vesicles. Front Immunol (2019) 10:448. doi: 10.3389/fimmu.2019.00448 30915085 PMC6423080

[45] Torralba D Baixauli F Villarroya-Beltri C Fernández-Delgado I Latorre-Pellicer A Acín-Pérez R. Priming of dendritic cells by DNA-containing extracellular vesicles from activated T cells through antigen-driven contacts. Nat Commun (2018) 9(1):2658. doi: 10.1038/s41467-018-05077-9 29985392 PMC6037695

[46] Koenig A Buskiewicz-Koenig IA. Redox activation of mitochondrial DAMPs and the metabolic consequences for development of autoimmunity. Antioxid Redox Signal (2022) 36(7-9):441–61. doi: 10.1089/ars.2021.0073 PMC8982130 35352943

[47] Sun YF Pi J Xu JF. Emerging role of exosomes in tuberculosis: from immunity regulations to vaccine and immunotherapy. Front Immunol (2021) 12:628973. doi: 10.3389/fimmu.2021.628973 33868247 PMC8047325

[48] Alvarez-Jiménez VD Leyva-Paredes K García-Martínez M Vázquez-Flores L García-Paredes VG Campillo-Navarro M. Extracellular vesicles released from *mycobacterium tuberculosis*-infected neutrophils promote macrophage autophagy and decrease intracellular mycobacterial survival. Front Immunol (2018) 9:272. doi: 10.3389/fimmu.2018.00272 29520273 PMC5827556

[49] Yuan Q Chen H Yang Y Fu Y Yi Z. miR-18a promotes Mycobacterial survival in macrophages via inhibiting autophagy by down-regulation of ATM. J Cell Mol Med (2020) 24(2):2004–12. doi: 10.1111/jcmm.14899 PMC6991191 31845528

[50] Athman JJ Sande OJ Groft SG Reba SM Nagy N Wearsch PA. *Mycobacterium tuberculosis* membrane vesicles inhibit T cell activation. J Immunol (2017) 198(5):2028–37. doi: 10.4049/jimmunol.1601199 PMC5322216 28122965

[51] Singh PP LeMaire C Tan JC Zeng E Schorey JS. Exosomes released from M. tuberculosis infected cells can suppress IFN-γ mediated activation of naïve macrophages. PloS One (2011) 6(4):e18564. doi: 10.1371/journal.pone.0018564 21533172 PMC3077381

[52] Iacomino G. miRNAs: the road from bench to bedside. Genes (Basel) (2023) 14(2):314. doi: 10.3390/genes14020314 36833241 PMC9957002

[53] Farina FM Hall IF Serio S Zani S Climent M Salvarani N. miR-128-3p is a novel regulator of vascular smooth muscle cell phenotypic switch and vascular diseases. Circ Res (2020) 126(12):e120–35. doi: 10.1161/CIRCRESAHA.120.316489 32216529

[54] Song Y Zhang C Zhang J Jiao Z Dong N Wang G. Localized injection of miRNA-21-enriched extracellular vesicles effectively restores cardiac function after myocardial infarction. Theranostics (2019) 9(8):2346–60. doi: 10.7150/thno.29945 PMC6531307 31149048

[55] Zhu Q Zhang Q Gu M Zhang K Xia T Zhang S. MIR106A-5p upregulation suppresses autophagy and accelerates Malignant phenotype in nasopharyngeal carcinoma. Autophagy (2021) 17(7):1667–83. doi: 10.1080/15548627.2020.1781368 PMC8354606 32627648

[56] Riahi Rad Z Riahi Rad Z Goudarzi H Goudarzi M Mahmoudi M Yasbolaghi Sharahi J. MicroRNAs in the interaction between host-bacterial pathogens: A new perspective. J Cell Physiol (2021) 236(9):6249–70. doi: 10.1002/jcp.30333 33599300

[57] Hill M Tran N. miRNA:miRNA interactions: A novel mode of miRNA regulation and its effect on disease. Adv Exp Med Biol (2022) 1385:241–57. doi: 10.1007/978-3-031-08356-3_9 36352217

[58] Sun Z Shi K Yang S Liu J Zhou Q Wang G. Effect of exosomal miRNA on cancer biology and clinical applications. Mol Cancer (2018) 17(1):147. doi: 10.1186/s12943-018-0897-7 30309355 PMC6182840

[59] Yu X Odenthal M Fries JW. Exosomes as miRNA carriers: formation-function-future. Int J Mol Sci (2016) 17(12):2028. doi: 10.3390/ijms17122028 27918449 PMC5187828

[60] Matsuyama H Suzuki HI. Systems and synthetic microRNA biology: from biogenesis to disease pathogenesis. Int J Mol Sci (2019) 21(1):132. doi: 10.3390/ijms21010132 31878193 PMC6981965

[61] Kilikevicius A Meister G Corey DR. Reexamining assumptions about miRNA-guided gene silencing. Nucleic Acids Res (2022) 50(2):617–34. doi: 10.1093/nar/gkab1256 PMC8789053 34967419

[62] Zhang D Yi Z Fu Y. Downregulation of miR-20b-5p facilitates *Mycobacterium tuberculosis* survival in RAW 264.7 macrophages via attenuating the cell apoptosis by Mcl-1 upregulation. J Cell Biochem (2019) 120(4):5889–96. doi: 10.1002/jcb.27874 30378171

[63] Zhan X Yuan W Zhou Y Ma R Ge Z. Small RNA sequencing and bioinformatics analysis of RAW264.7-derived exosomes after *Mycobacterium Bovis Bacillus Calmette-Guérin* infection. BMC Genomics (2022) 23(1):355. doi: 10.1186/s12864-022-08590-w 35525953 PMC9080156

[64] Kumar R Sahu SK Kumar M Jana K Gupta P Gupta UD. MicroRNA 17-5p regulates autophagy in *Mycobacterium tuberculosis*-infected macrophages by targeting Mcl-1 and STAT3. Cell Microbiol (2016) 18(5):679–91. doi: 10.1111/cmi.12540 26513648

[65] Kaushik AC Wu Q Lin L. Exosomal ncRNAs profiling of mycobacterial infection identified miRNA-185-5p as a novel biomarker for tuberculosis. Brief Bioinform (2021) 22(6):bbab210. doi: 10.1093/bib/bbab210 34169968

[66] Tu H Yang S Jiang T Wei L Shi L Liu C. Elevated pulmonary tuberculosis biomarker miR-423-5p plays critical role in the occurrence of active TB by inhibiting autophagosome-lysosome fusion. Emerg Microbes Infect (2019) 8(1):448–60. doi: 10.1080/22221751.2019.1590129 PMC6455132 30898038

[67] Lyu L Zhang X Li C Yang T Wang J Pan L. Small RNA profiles of serum exosomes derived from individuals with latent and active tuberculosis. Front Microbiol (2019) 10:1174. doi: 10.3389/fmicb.2019.01174 31191492 PMC6546874

[68] Alipoor SD Tabarsi P Varahram M Movassaghi M Dizaji MK Folkerts G. Serum exosomal miRNAs are associated with active pulmonary tuberculosis. Dis Markers (2019) 2019:1907426. doi: 10.1155/2019/1907426 30886653 PMC6388314

[69] Wang Y Xu YM Zou YQ Lin J Huang B Liu J. Identification of differential expressed PE exosomal miRNA in lung adenocarcinoma, tuberculosis, and other benign lesions. Med (Baltimore) (2017) 96(44):e8361. doi: 10.1097/MD.0000000000008361 PMC5682784 29095265

[70] Zhang X Bao L Yu G Wang H. Exosomal miRNA-profiling of pleural effusion in lung adenocarcinoma and tuberculosis. Front Surg (2023) 9:1050242. doi: 10.3389/fsurg.2022.1050242 36684253 PMC9852630

[71] Guio H Aliaga-Tobar V Galarza M Pellon-Cardenas O Capristano S Gomez HL. Comparative profiling of circulating exosomal small RNAs derived from Peruvian patients with tuberculosis and pulmonary adenocarcinoma. Front Cell Infect Microbiol (2022) 12:909837. doi: 10.3389/fcimb.2022.909837 35846752 PMC9280157

[72] Carranza C Herrera MT Guzmán-Beltrán S Salgado-Cantú MG Salido-Guadarrama I Santiago E. A Dual Marker for Monitoring MDR-TB Treatment: Host-Derived miRNAs and M. tuberculosis-Derived RNA Sequences in Serum. Front Immunol (2021) 12:760468. doi: 10.3389/fimmu.2021.760468 34804048 PMC8600136

[73] Barry SE Ellis M Yang Y Guan G Wang X Britton WJ. Identification of a plasma microRNA profile in untreated pulmonary tuberculosis patients that is modulated by anti-mycobacterial therapy. J Infect (2018) 77(4):341–8. doi: 10.1016/j.jinf.2018.03.006 29746939

[74] Patop IL Wüst S Kadener S. Past, present, and future of circRNAs. EMBO J (2019) 38(16):e100836. doi: 10.15252/embj.2018100836 31343080 PMC6694216

[75] Wang Y Liu J Ma J Sun T Zhou Q Wang W. Exosomal circRNAs: biogenesis, effect and application in human diseases. Mol Cancer (2019) 18(1):116. doi: 10.1186/s12943-019-1041-z 31277663 PMC6610963

[76] Kour B Gupta S Singh R Sophiarani Y Paul P. Interplay between circular RNA, microRNA, and human diseases. Mol Genet Genomics (2022) 297(2):277–86. doi: 10.1007/s00438-022-01856-8 35084582

[77] Zhou WY Cai ZR Liu J Wang DS Ju HQ Xu RH. Circular RNA: metabolism, functions and interactions with proteins. Mol Cancer (2020) 19(1):172. doi: 10.1186/s12943-020-01286-3 33317550 PMC7734744

[78] Luo HL Peng Y Luo H Zhang JA Liu GB Xu H. Circular RNA hsa_circ_0001380 in peripheral blood as a potential diagnostic biomarker for active pulmonary tuberculosis. Mol Med Rep (2020) 21(4):1890–6. doi: 10.3892/mmr.2020.10992 PMC7057807 32319627

[79] Wang J Li Y Wang N Wu J Ye X Jiang Y. Functions of exosomal non-coding RNAs to the infection with Mycobacterium tuberculosis. Front Immunol (2023) 14:1127214. doi: 10.3389/fimmu.2023.1127214 37033928 PMC10073540

[80] Yi Z Gao K Li R Fu Y. Dysregulated circRNAs in plasma from active tuberculosis patients. J Cell Mol Med (2018) 22(9):4076–84. doi: 10.1111/jcmm.13684 PMC6111852 29961269

[81] Huang Z Su R Qing C Peng Y Luo Q Li J. Plasma Circular RNAs hsa_circ_0001953 and hsa_circ_0009024 as Diagnostic Biomarkers for Active Tuberculosis. Front Microbiol (2018) 9:2010. doi: 10.3389/fmicb.2018.02010 30214434 PMC6126419

[82] Huang Z Su R Yao F Peng Y Luo Q Li J. Circulating circular RNAs hsa_circ_0001204 and hsa_circ_0001747 act as diagnostic biomarkers for active tuberculosis detection. Int J Clin Exp Pathol (2018) 11(2):586–94. PMC6958007 31938144

[83] Liu H Lu G Wang W Jiang X Gu S Wang J. A panel of circRNAs in the serum serves as biomarkers for *mycobacterium tuberculosis* infection. Front Microbiol (2020) 11:1215. doi: 10.3389/fmicb.2020.01215 32582119 PMC7296121

[84] Mumtaz PT Taban Q Dar MA Mir S Haq ZU Zargar SM. Deep Insights in Circular RNAs: from biogenesis to therapeutics. Biol Proced Online (2020) 22:10. doi: 10.1186/s12575-020-00122-8 32467674 PMC7227217

[85] Zhang Q Wang W Zhou Q Chen C Yuan W Liu J. Roles of circRNAs in the tumour microenvironment. Mol Cancer (2020) 19(1):14. doi: 10.1186/s12943-019-1125-9 31973726 PMC6977266

[86] Yuan Q Wen Z Yang K Zhang S Zhang N Song Y. Identification of key circRNAs related to pulmonary tuberculosis based on bioinformatics analysis. BioMed Res Int (2022) 2022:1717784. doi: 10.1155/2022/1717784 35419455 PMC9001091

[87] Yi XH Zhang B Fu YR Yi ZJ. STAT1 and its related molecules as potential biomarkers in *Mycobacterium tuberculosis* infection. J Cell Mol Med (2020) 24(5):2866–78. doi: 10.1111/jcmm.14856 PMC7077527 32048448

[88] Zhang X Zhang Q Wu Q Tang H Ye L Zhang Q. Integrated analyses reveal hsa_circ_0028883 as a diagnostic biomarker in active tuberculosis. Infect Genet Evol (2020) 83:104323. doi: 10.1016/j.meegid.2020.104323 32305357

[89] Zhuang ZG Zhang JA Luo HL Liu GB Lu YB Ge NH. The circular RNA of peripheral blood mononuclear cells: Hsa_circ_0005836 as a new diagnostic biomarker and therapeutic target of active pulmonary tuberculosis. Mol Immunol (2017) 90:264–72. doi: 10.1016/j.molimm.2017.08.008 28846924

[90] Huang ZK Yao FY Xu JQ Deng Z Su RG Peng YP. Microarray expression profile of circular RNAs in peripheral blood mononuclear cells from active tuberculosis patients. Cell Physiol Biochem (2018) 45(3):1230–40. doi: 10.1159/000487454 29448254

[91] Huang Z Su R Deng Z Xu J Peng Y Luo Q. Identification of differentially expressed circular RNAs in human monocyte derived macrophages response to *Mycobacterium tuberculosis* infection. Sci Rep (2017) 7(1):13673. doi: 10.1038/s41598-017-13885-0 29057952 PMC5651861

[92] Li Y Zheng Q Bao C Li S Guo W Zhao J. Circular RNA is enriched and stable in exosomes: a promising biomarker for cancer diagnosis. Cell Res (2015) 25(8):981–4. doi: 10.1038/cr.2015.82 PMC4528056 26138677

[93] Zang J Lu D Xu A. The interaction of circRNAs and RNA binding proteins: An important part of circRNA maintenance and function. J Neurosci Res (2020) 98(1):87–97. doi: 10.1002/jnr.24356 30575990

[94] Giri PK Kruh NA Dobos KM Schorey JS. Proteomic analysis identifies highly antigenic proteins in exosomes from M. tuberculosis-infected and culture filtrate protein-treated macrophages. Proteomics (2010) 10(17):3190–202. doi: 10.1002/pmic.200900840 PMC3664454 20662102

[95] Lee J Kim SH Choi DS Lee JS Kim DK Go G. Proteomic analysis of extracellular vesicles derived from Mycobacterium tuberculosis. Proteomics (2015) 15(19):3331–7. doi: 10.1002/pmic.201500037 26201501

[96] Layre E. Trafficking of *mycobacterium tuberculosis* envelope components and release within extracellular vesicles: host-pathogen interactions beyond the wall. Front Immunol (2020) 11:1230. doi: 10.3389/fimmu.2020.01230 32765485 PMC7378356

[97] Diaz G Wolfe LM Kruh-Garcia NA Dobos KM. Changes in the Membrane-Associated Proteins of Exosomes Released from Human Macrophages after *Mycobacterium tuberculosis* Infection. Sci Rep (2016) 6:37975. doi: 10.1038/srep37975 27897233 PMC5126699

[98] Kruh-Garcia NA Wolfe LM Chaisson LH Worodria WO Nahid P Schorey JS. Detection of *Mycobacterium tuberculosis* peptides in the exosomes of patients with active and latent M. tuberculosis infection using MRM-MS. PloS One (2014) 9(7):e103811. doi: 10.1371/journal.pone.0103811 25080351 PMC4117584

[99] Zhang M Xie Y Li S Ye X Jiang Y Tang L. Proteomics analysis of exosomes from patients with active tuberculosis reveals infection profiles and potential biomarkers. Front Microbiol (2022) 12:800807. doi: 10.3389/fmicb.2021.800807 35069505 PMC8770970

[100] Mehaffy C Kruh-Garcia NA Graham B Jarlsberg LG Willyerd CE Borisov A. Identification of *mycobacterium tuberculosis* peptides in serum extracellular vesicles from persons with latent tuberculosis infection. J Clin Microbiol (2020) 58(6):e00393–20. doi: 10.1128/JCM.00393-20 PMC7269374 32245831

[101] Huang C Pan L Shen X Tian H Guo L Zhang Z. Hsp16.3 of *mycobacterium tuberculosis* in exosomes as a biomarker of tuberculosis. Eur J Clin Microbiol Infect Dis (2021) 40(11):2427–30. doi: 10.1007/s10096-021-04246-x 33893878

[102] Biadglegne F Schmidt JR Engel KM Lehmann J Lehmann RT Reinert A. *Mycobacterium tuberculosis* affects protein and lipid content of circulating exosomes in infected patients depending on tuberculosis disease state. Biomedicines (2022) 10(4):783. doi: 10.3390/biomedicines10040783 35453532 PMC9025801

[103] García-Martínez M Vázquez-Flores L Álvarez-Jiménez VD Castañeda-Casimiro J Ibáñez-Hernández M Sánchez-Torres LE. Extracellular vesicles released by J774A.1 macrophages reduce the bacterial load in macrophages and in an experimental mouse model of tuberculosis. Int J Nanomed (2019) 14:6707–19. doi: 10.2147/IJN.S203507 PMC6708438 31692512

[104] Dahiya B Khan A Mor P Kamra E Singh N Gupta KB. Detection of Mycobacterium tuberculosis lipoarabinomannan and CFP-10 (Rv3874) from urinary extracellular vesicles of tuberculosis patients by immuno-PCR. Pathog Dis (2019) 77(5):ftz049. doi: 10.1093/femspd/ftz049 31549171

[105] Du Y Xin H Cao X Liu Z He Y Zhang B. Association between plasma exosomes S100A9/C4BPA and latent tuberculosis infection treatment: proteomic analysis based on a randomized controlled study. Front Microbiol (2022) 13:934716. doi: 10.3389/fmicb.2022.934716 35935235 PMC9355536

[106] Li W Li C Zhou T Liu X Liu X Li X. Role of exosomal proteins in cancer diagnosis. Mol Cancer (2017) 16(1):145. doi: 10.1186/s12943-017-0706-8 28851367 PMC5576100

[107] Hu C Jiang W Lv M Fan S Lu Y Wu Q. Potentiality of exosomal proteins as novel cancer biomarkers for liquid biopsy. Front Immunol (2022) 13:792046. doi: 10.3389/fimmu.2022.792046 35757760 PMC9218252

[108] Kiran D Podell BK Chambers M Basaraba RJ. Host-directed therapy targeting the *Mycobacterium tuberculosis* granuloma: a review. Semin Immunopathol (2016) 38(2):167–83. doi: 10.1007/s00281-015-0537-x PMC4779125 26510950

[109] Augenstreich J Briken V. Host cell targets of released lipid and secreted protein effectors of mycobacterium tuberculosis. Front Cell Infect Microbiol (2020) 10:595029. doi: 10.3389/fcimb.2020.595029 33194845 PMC7644814

[110] Ishikawa E Mori D Yamasaki S. Recognition of mycobacterial lipids by immune receptors. Trends Immunol (2017) 38(1):66–76. doi: 10.1016/j.it.2016.10.009 27889398

[111] Han YS Chen JX Li ZB Chen J Yi WJ Huang H. Identification of potential lipid biomarkers for active pulmonary tuberculosis using ultra-high-performance liquid chromatography-tandem mass spectrometry. Exp Biol Med (Maywood) (2021) 246(4):387–99. doi: 10.1177/1535370220968058 PMC7885049 33175608

[112] Wu M Yang Q Yang C Han J Lui H Qiao L. Characteristics of plasma exosomes in drug-resistant tuberculosis patients. Tuberculosis (Edinb) (2023) 141:102359. 37329682 10.1016/j.tube.2023.102359

[113] Krug S Parveen S Bishai WR. Host-directed therapies: Modulating inflammation to treat tuberculosis. Front Immunol (2021) 12:660916. doi: 10.3389/fimmu.2021.660916 33953722 PMC8089478

[114] Zhang W Jiang X Bao J Wang Y Liu H Tang L. Exosomes in pathogen infections: A bridge to deliver molecules and link functions. Front Immunol (2018) 9:90. doi: 10.3389/fimmu.2018.00090 29483904 PMC5816030

[115] Kim JS Kim YR Yang CS. Host-directed therapy in tuberculosis: Targeting host metabolism. Front Immunol (2020) 11:1790. doi: 10.3389/fimmu.2020.01790 32903583 PMC7438556

[116] Yu D Li Y Wang M Gu J Xu W Cai H. Exosomes as a new frontier of cancer liquid biopsy. Mol Cancer (2022) 21(1):56. doi: 10.1186/s12943-022-01509-9 35180868 PMC8855550

